# Mapping Forest Cover in Northeast China from Chinese HJ-1 Satellite Data Using an Object-Based Algorithm

**DOI:** 10.3390/s18124452

**Published:** 2018-12-16

**Authors:** Chunying Ren, Bai Zhang, Zongming Wang, Lin Li, Mingming Jia

**Affiliations:** 1 Key Laboratory of Wetland Ecology and Environment, Northeast Institute of Geography and Agroecology, Chinese Academy of Sciences, Changchun 130102, China; renchy@iga.ac.cn (C.R.); zongmingwang@iga.ac.cn (Z.W.); jiamingming@iga.ac.cn (M.J.); 2 Department of Earth Sciences, Indiana University-Purdue University, Indianapolis, IN 46202, USA; ll3@iupui.edu

**Keywords:** forest mapping, Northeast China, HJ-1 imagery, object-oriented classification, MCD12Q1, GlobCover

## Abstract

Forest plays a significant role in the global carbon budget and ecological processes. The precise mapping of forest cover can help significantly reduce uncertainties in the estimation of terrestrial carbon balance. A reliable and operational method is necessary for a rapid regional forest mapping. In this study, the goal relies on mapping forest and subcategories in Northeast China through the use of high spatio-temporal resolution HJ-1 imagery and time series vegetation indices within the context of an object-based image analysis and decision tree classification. Multi-temporal HJ-1 images obtained in a single year provide an opportunity to acquire phenology information. By analyzing the difference of spectral and phenology information between forest and non-forest, forest subcategories, decision trees using threshold values were finally proposed. The resultant forest map has a high overall accuracy of 0.91 ± 0.01 with a 95% confidence interval, based on the validation using ground truth data from field surveys. The forest map extracted from HJ-1 imagery was compared with two existing global land cover datasets: GlobCover 2009 and MCD12Q1 2009. The HJ-1-based forest area is larger than that of MCD12Q1 and GlobCover and more closely resembles the national statistics data on forest area, which accounts for more than 40% of the total area of the Northeast China. The spatial disagreement primarily occurs in the northern part of the Daxing’an Mountains, Sanjiang Plain and the southwestern part of the Songliao Plain. The compared result also indicated that the forest subcategories information from global land cover products may introduce large uncertainties for ecological modeling and these should be cautiously used in various ecological models. Given the higher spatial and temporal resolution, HJ-1-based forest products could be very useful as input to biogeochemical models (particularly carbon cycle models) that require accurate and updated estimates of forest area and type.

## 1. Introduction

Forest covers approximately 31% of the global land surface [1,2] and dramatic changes in forested areas have attracted much attention in the past few decades due to their strong influence on regional climate, water and carbon cycles, as well as biodiversity [3,4]. Timely and accurate information on forest cover from global to regional scales is needed for natural resource management, carbon cycle studies, and biogeochemistry, hydrology, and climate modeling [5].

Northeast China harbors the largest relatively contiguous area of forested land in contemporary China [6], which is composed of different types of forest, including evergreen needleleaf forest, deciduous needleleaf forest, deciduous broadleaf forest, and mixed forest [7]. This region has experienced dramatic deforestation due to agricultural activities, urbanization, and water project constructions [8,9]. According to the national survey of forest resources, the area of mature forest contracted 49.0% in the period 1981–1988; then, in the following 10 years (1988–1998), 0.61 million ha of forest area further disappeared, accounting for approximately 60.0% of the entire country’s total mature forest area [5]. According to the 6th National Continuous Forest Inventory in 2003, the forested area of Northeast China increased by 12.2 million ha compared to the forested area in 1950. In the forest provinces of Northeast China, the declined forest was almost entirely restored during this same period [10]. Northeast China is a pilot region for the Grain for Green Project, the Logging Ban Project, and other ecological restoration projects that have, to some extent, been successful in meeting their objectives [11,12]. However, the forest-contracting trend still exists [13,14]. The lack of readily accessible, peer-reviewed data sources results in the statistics on forest cover in Northeast China cited by scientific publications that varied widely. Additionally, the reliability of forest trend data analysis is uncertain due to inconsistencies among study periods or the use of different data sources by researchers. It is essential for government managers and researchers to obtain accurate and updated information on forest areas and variation at the regional scale.

Remote sensing has significantly contributed to the detection of forest extent, types, and changes at regional and global scales [15,16]. A number of earlier studies mapped forest in Northeast China using remote sensing data, including the NOAA/AVHRR data [17], SPOT-4 VEGETATION composite data [5] and MODIS datasets [18,19]. Additionally, many global land-cover products are available at different spatial resolutions, ranging from 300 m to 1 km, including UMD 1992/1993 (1-km resolution), GLC2000 (1-km resolution), GlobCover 2004/2009 (300-m resolution), and MODIS Land Cover Type (500 m resolution) [20]. The derivation of forest areal statistics with these global products for Northeast China requires a number of GIS operations such as re-projection, mosaicking, sub-setting, and recoding. Given that the overall classification accuracies of global products range between 65% and 75% [21], coarse resolution datasets fail to provide reliable area estimates of forest extent and change [22]. Landsat TM/ETM+ imagery has been widely used to map forest areas in Northeast China [23,24,25,26]. Because of frequent cloud cover and the long re-visit time (16 days) of Landsat, it is difficult to obtain cloud-free images over the entirety of Northeast China during a certain period. Furthermore, successive acquisition of the Landsat data in a single year is very difficult and then temporal or phenological information cannot be incorporated into the procedure for forest mapping, although phenological information is very important for improving vegetation cover classification [5,27]. The Chinese satellite constellation HJ-1 was launched in 2008. Due to the fine spatial resolution (30 m), large swath (700 km), and short revisit cycle (2 days), it has been used to map saltmarsh and species [28], rice growth parameters [29], and land cover [30], and it is expected to be an important data source for rapid, large area land cover information extraction.

In the most recent decades, there are various remote sensing-based methodologies that have been widely used to obtain the extent and subcategories of forest, including conventional supervised and unsupervised classification [31,32], the phenology-based method [19], and object-based classification [33]. Using these methods, classification accuracies of 70–90% for forest cover or types were reported. The object-based classification is especially becoming more popular compared with traditional pixel-based image analysis [34], because it allows for segmentation, attribution, classification, and establishment of relationships among defined objects that are not possible in pixel-based analysis [35]. The advantages of object-based classification also include overcoming salt-and-pepper effects [36] and providing geo-information that can be immediately stored into Geographical Information System (GIS) databases [37]. Furthermore, many studies revealed that the object-based classification provided higher accuracy than the pixel-based classification approaches [38,39,40]. Some research that incorporated other sources of remotely sensed data (such as LiDAR and Hyperspectral data) into the object-based forest classification process has been reported [41,42,43]. However, little research has explored the combination of phenology analysis and the object-based approach, which may increase the accuracy of classification and contribute towards operational large-scale forest mapping.

Based on the necessity of forest information in Northeast China and the existing challenges in large-scale forest mapping in high resolution, the innovative goal tackled in this paper relies on the use of multi-temporal HJ-1 CCD imagery, within the context of an object-based classification approach, to map the extent and subcategories of forest through a decision tree classifier. By taking the spatio-temporal advantages of HJ-1 CCD data, a hierarchical classification approach coupled with time series Vegetation Indices and DEM information was developed to map forest in Northeast China. The other specific objective of this study is to compare the HJ-1-based forest map with two global land cover datasets (GlobCover 2009 and MODIS MCD12Q1) produced for similar years, which will help explain the error and uncertainty of forest monitoring at large spatial scales from these three datasets. The results from this study can support forest management and regional sustainable development.

## 2. Materials and Methods

### 2.1. Study Area

**Figure 1:**
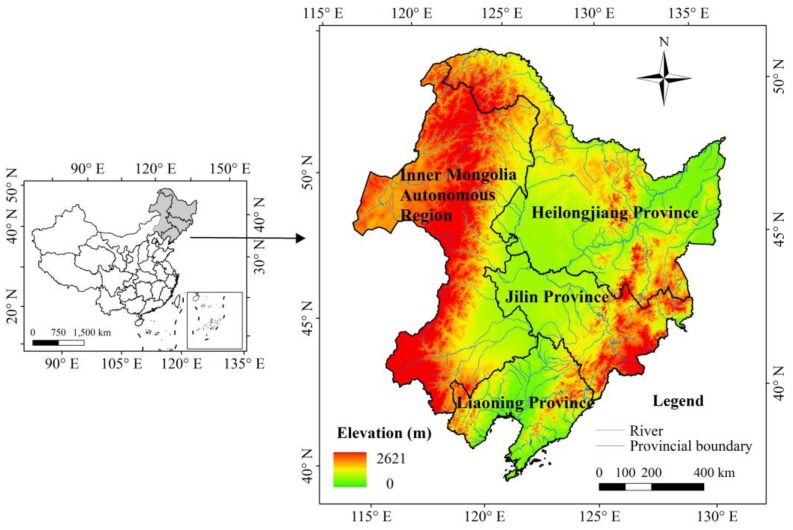
Location and elevation of Northeast China.

Northeast China includes the provinces of Heilongjiang (HLJ), Jilin (JL), Liaoning (LN) and the eastern part of the Inner Mongolia Autonomous Region, encompassing 124 × 10^4^ km^2^ (Figure 1). The region is influenced by the high latitude East Asia monsoon. The climate changes from a warm temperate, temperate to cool temperate from south to north, and from humid, semi-humid to semi-arid from east to west. The annual mean temperature ranges from 11.5 to −4 °C and annual precipitation varies from 1100 mm to 250 mm [44]. The forest region of Northeast China is primarily distributed in the Changbai Mountains, Daxing’an Mountains and Xiaoxing’an Mountains, whose elevations predominantly range between 1000 and 2000 m. The forest types vary from deciduous broadleaf, needleleaf, and broadleaf mixed forest, to boreal forest with increasing latitude. The major species include Dahurian larch (*Larix gmelini*), Korean pine (*Pinus koraiensis*), spruce (*Picea koraiensis*), fir (*Abies nephrolepsis*), basswood (*Tilia amurensis*), mongolian oak (*Quercus mongolica*), birch (*Betual platyphyua*) and aspen (*populous davidiana*).

### 2.2. HJ-1 Satellite Images and Preprocessing

The satellite constellation HJ-1 was designed for environmental and disaster monitoring and forecasting, and includes three satellites: HJ-1A, HJ-1B, and HJ-1C. HJ-1A and HJ-1B carry two identical CCD sensors onboard, each with 30-m spatial resolution [45]. The HJ-1 CCDs have three visible bands (430–520 nm, 520–600 nm, and 630–690 nm) and one near-infrared band (760–900 nm), which are equivalent to Landsat TM bands 1, 2, 3, and 4, respectively. The two satellite CCD sensors acquire images with a swath of approximately 700 km and have a revisiting time of 48 h. Therefore, the HJ-1 CCDs can provide images that encompass all of China’s territory every two or three days. The HJ-1 CCD data have the potential to be an important data source for forest mapping, as they are freely available to the public at the official websites of the Ministry of Environmental Protection’s Satellite Environment Center (www.secmep.cn) or China’s Center for Resources Satellite Data and Application (http://www.cresda.com).

A total of 96 scenes of HJ-1 CCD images was acquired to cover the entirety of Northeast China for the period from late April to November of 2010. At least three scenes from spring to fall were required to cover the same forest region, thus ensuring the detection of seasonal changes of different forest types, less cloud cover, and an absence of significant aerosols. Data preprocessing, including geometric correction, atmospheric correction, and radiometric calibration, was performed to create a high-quality image dataset. Although HJ-1 CCD data have a spatial reference, the spatial error is approximately 200–300 m larger than that for geo-referenced Landsat TM imagery. Therefore, the Landsat TM L1T products were used as the base images downloaded from the USGS website (http://glovis.usgs.gov/). All HJ-1 CCD images were automatically geometrically corrected according to the image-to-image registration method integrated into the Auto Sync module of the ERDAS software version 9.2 and the RMS errors of the control points were limited to within two pixels. In the areas with rough terrain, ASTER GDEM (30-m resolution) obtained from the USGS website (http://gdex.cr.usgs.gov/gdex/) was used for image ortho-rectification. All corrected images were projected onto an Albers projection with a WGS-84 ellipsoid. The FLAASH module in the ENVI software version 5.3 [46] was applied to alleviate the atmospheric effects on the HJ-1 CCD images. The atmospheric water vapor contents were estimated with one of the MODIS/Terra products (MOD05) [47]. The other parameters were constructed based on the acquisition time and location for the imagery and other ancillary data. The DN values of the HJ-1 CCD images were transferred into the top of atmosphere (TOA) radiance with given spectral response functions under the platform of IDL in the ENVI software.

Time series of vegetation indices can capture the indices variation in a specific period, and have proven valuable for forest cover classification [48]. In this study, the NDVI, NDWI, RVI and EVI were calculated for each of the HJ-1 composite images and were used in the forest classification. These indices are defined by the following equations:

NDVI_HJ_ = (B4 − B3)/(B4 + B3) (1)

NDWI_HJ_ = (B2 − B4)/(B2 + B4) (2)

RVI_HJ_ = B4/B3 (3)

EVI_HJ_ = 2.5 × (B4 − B3)/(B4 + 6 × B3 − 7.5 × B1 + 1) (4)

where B1, B2, B3 and B4 represent band 1, band 2, band 3, and band 4 of the HJ-1 CCD images, respectively. These VI images and HJ-1 CCD composite images for the same region at different seasons were input to the eCognition Developer 8.64 [49] for land cover classification.

### 2.3. Classification System

The land cover classification system used in this study was adapted from the top-level land categories of the Intergovernmental Panel on Climate Change (IPCC) [50], including forest, cropland, grassland, wetland, water, settlements, and other land. To create detailed forest types and ensure the consistency of this forest classification with the other land cover data products to be compared in this study, the forest was further classified into six categories: evergreen needleleaf forest, evergreen broadleaf forest, deciduous needleleaf forest, deciduous broadleaf forest, mixed forest, and shrubland.

### 2.4. Ground Truth Data for Training and Validation

In this study, ground truth data for training were collected by nine groups of researchers in vehicles traversing approximately the entirety of Northeast China from June to August 2011. Before each field trip, the geographic distribution of sampling points and proximity routes for each traverse were prepared to ensure successful data collection. The location of each sampling point was documented using a global positioning system (GPS), with errors of less than 10 m. A vector file of ground survey points with their location (longitude and latitude), land cover types, and photos was created using ArcGIS [51]. In total, 2938 points were selected as training samples for the forest classification, including 59 evergreen needleleaf forest points, 210 deciduous needleleaf forest points, 295 deciduous broadleaf forest points, 200 mixed forest points, 102 shrubland points, and 2072 non-forest points.

**Figure 2:**
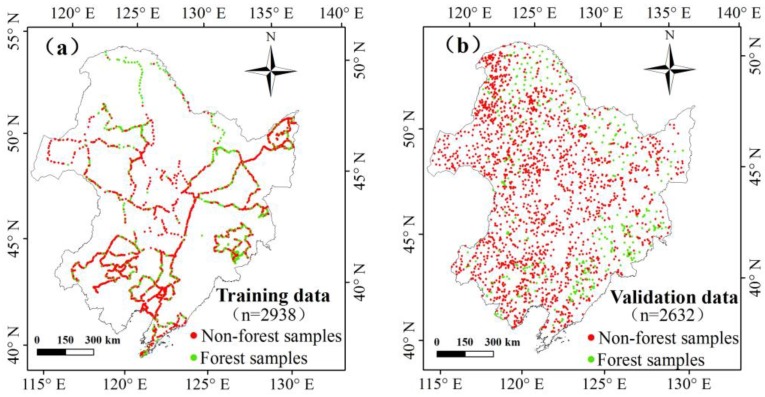
Location of training data (**a**) and validation data (**b**) in Northeast China.

The accuracy of the HJ-1-based forest map was assessed using validation data acquired from the field trips in 2011. The field acquisition approach for collecting the validation data was the same as that for the training data; however, the validation data were provided by individual provincial environmental protection departments of Northeast China. To ensure the consistency of the land cover identification undertaken by different participants, a standard protocol of defining each class and the field sampling procedure was created before each field trip. All field trip participants were required to follow a standard protocol in the documentation of their field sites and landscapes using digital cameras and handheld GPS receivers. In total, 2632 points were obtained, including 685 forest points and 1947 non-forest points. The forest validation points included 110 evergreen needleleaf forest points, 133 deciduous needleleaf forest points, 254 deciduous broadleaf forest points, 118 mixed forest points, and 50 shrubland points for the accuracy assessment of the forest classification. The spatial distribution of the ground truth data for training and validation data are shown in Figure 2a,b.

### 2.5. Forest Classification Method

A hierarchical classification approach was developed to map forest cover and detailed forest types in Northeast China. The overall idea of this method is to improve the forest mapping accuracy over large areas by using the spectral information, phenology information, and DEM data, within the context of an object-based classification approach. The approach consists of three major steps. The first step is to partition a large area into several small mapping zones. Forest classification is then conducted in each zone independently. The second step is to create image objects by means of multi-resolution segmentation. At last, forest area and types are classified by coupling time series HJ-1 data, DEM, expert knowledge, etc.

#### 2.5.1. Spatial Partitioning

**Figure 3:**
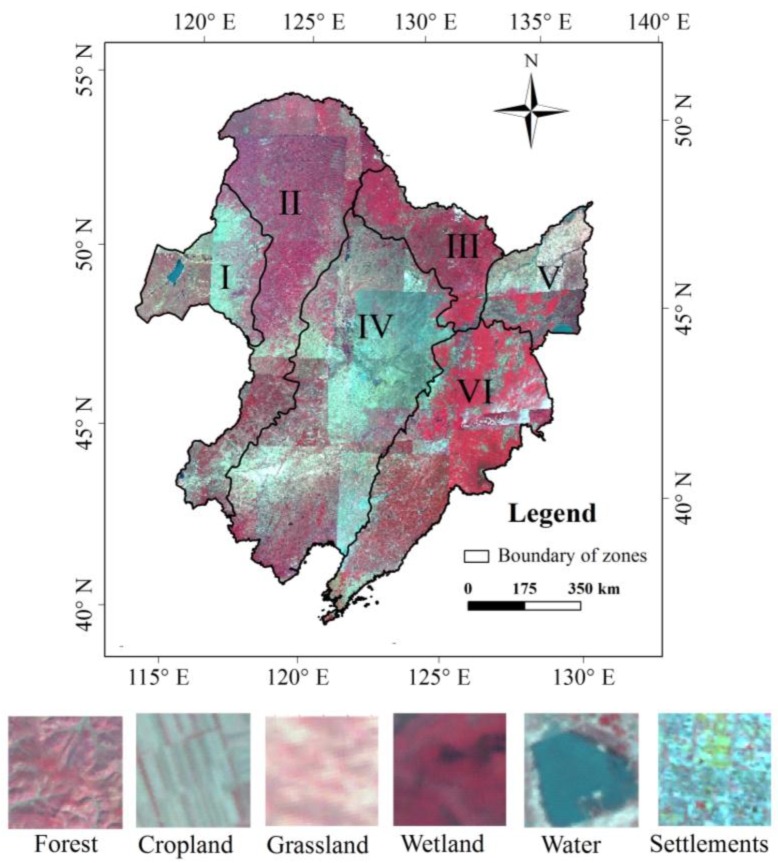
False color images, typical land cover types and zones based on climate and vegetation for the forest classification of Northeast China (I: Hulun Buir region; II: Daxing’an Mountains; III: Xiaoxing’an Mountains; IV: Songliao Plain; V: Sanjiang Plain; VI: Changbai Mountains).

The common practice of classification is to partition the large area into several small mapping zones that have similar climate and vegetation [52]. In this study, Northeast China was divided into six sub-regions based on the vegetation map of China [53] and the ecological functional region maps of China [54], including the Hulun Buir region, Daxing’an Mountains, Xiaoxing’an Mountains, Songliao Plain, Sanjiang Plain, and Changbai Mountains (Figure 3). Forest mapping is then conducted in each sub-region independently. After classifying imagery for the entire region, the results were mosaicked into a forest map of Northeast China.

#### 2.5.2. Multi-Resolution Segmentation

Multi-resolution segmentation was employed to the whole study area by means of the eCognition Developer 9.2.1 [49]. Image objects were created by segmenting an image into groups of homogeneous pixels so that the variability within individual objects was minimized [55]. The size of the object is controlled by a threshold that is defined by the scale, shape, and compactness parameters specified by users in a local optimization procedure. The scale parameter determines the maximum size of the created objects. Users can apply weights from 0 to 1 to the shape and compactness factors to determine objects at a certain level of scale. Two levels of the segmentation scale were used in this study (Table 1). The first level was used to discriminate forest and non-forest class and then the non-forest mask was created, the second level was utilized to produce a new image with the new grouping of pixels for classifying subcategories of forest. After a “trial and error” process for testing the segmentation parameters, a satisfactory match between image objects and landscape features was achieved when the scale parameter was set to 30 at level 1 and 10 at level 2. Forest objects are irregularly shaped, and less weight was therefore assigned to the shape than to the spectral homogeneity (the shape factor was set to 0.3 at the level 1and 0.1 at level 2). The compactness parameter was set at 0.6 to give a little higher weight to compactness than smoothness at both two levels.

#### 2.5.3. Object-Based Decision Tree Algorithm

**Figure 4:**
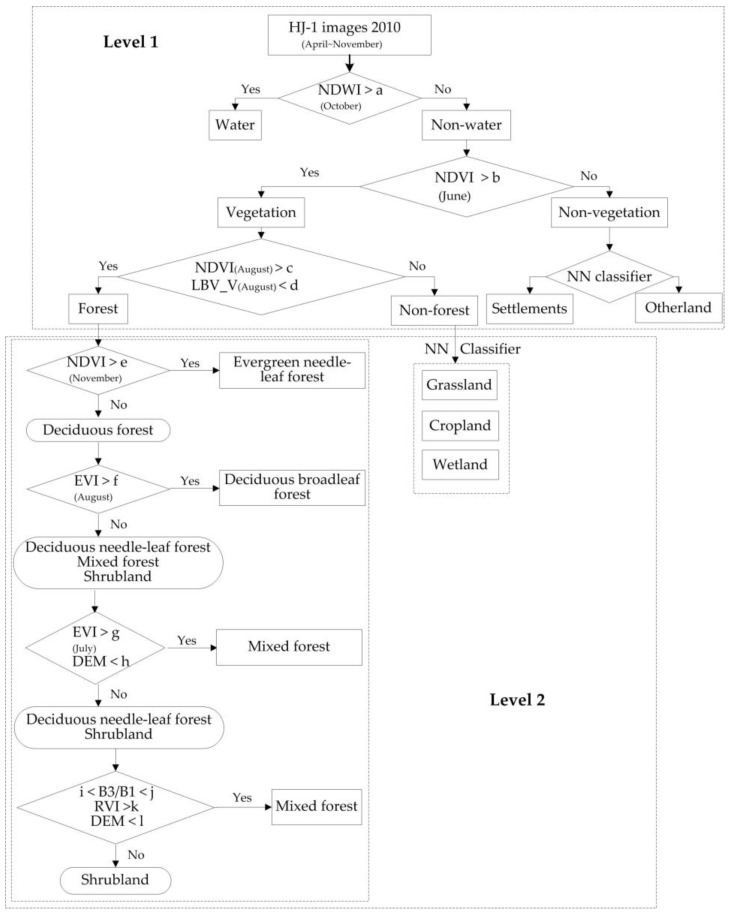
Forest classification based on multi-temporal images and an object-oriented method (a, b, c, d, e, f, g, h, i, j, k, l represents threshold at each decision tree node, respectively).

The overall idea of this approach is to develop a simple decision tree algorithm that uses threshold values from variables, which include the HJ-1 image bands 1–4, NDVI, NDWI, RVI, EVI, DEM and LBV transformation images (Figure 4). Thresholds in the decision tree rules were determined according to a statistical analysis of training areas created from the ground truth data. At first, water areas were distinguished using the Normalized Difference Water Index (NDWI) derived from an intermediate season. Non-water areas were then further classified into vegetation and non-vegetation classes using the Normalized Difference Vegetation Index (NDVI) derived from plant-growing seasons. The non-vegetation class included settlements and other land, distinguished using nearest neighbor (NN) classifiers. The bulk vegetation class was then re-classified into forest and non-forest classes (including cropland, grassland and wetland). The LBV transformation was proposed by Zeng [56]. Among the three transformation images, L image reflects the general radiance level, B image represents the visible–infrared radiation balance, and V image refers to the band radiance variation vector (direction and speed). The L, B, and V images were generated, and it was found that forest tended to have a relatively lower V component than other vegetation. Therefore, the V component, and NDVI were combined to separate the forest from non-forest classes. Forested areas were further classified into various forest types under the segmentation level 2 using multi-temporal HJ-1 composite images, vegetation index images, and DEM. The mean value of EVI and NDVI for different forest types monthly was calculated within the context of objects. By analyzing the multi-temporal EVI and NDVI of various forests, NDVI conclusively has advantages for separating evergreen and deciduous forest in early spring and winter. EVI is more sensitive to deciduous forest and mixed forest than NDVI in the summer. In addition, the distribution of needleleaf and broadleaf mixed forest has elevation limits in mountain regions, i.e., lower than 1000 m in the Changbai Mountains, lower than 900 m in the Zhangguangcai Mountains, and lower than 700 m in the Xiaoxing’an Mountains [57]. The EVI, NDVI, DEM, and expert knowledge were applied to classify deciduous broadleaf forest, deciduous needle-leaf forest, and mixed forest. Shrubland always mixes with deciduous broadleaf forest or mixed forest. Based on the relevant literature and vegetation maps of China, the total shrubland area in Northeast China is relatively smaller than other forest types. According to the training samples for shrubland, the ratio of band 3 and band 1, the RVI, and DEM were used to extract the shrubland group from deciduous broadleaf forest or mixed forest.

### 2.6. Accuracy Assessment of the HJ-1-Based Forest Map in 2010

To assess the accuracy of forest classification, a confusion matrix was adopted to measure the agreement between our classification result and validation data. The user’s accuracy, producer’s accuracy, and overall accuracy were calculated. The overall accuracy represents the percentage of the points correctly identified [58]. The user’s accuracy demonstrates the likelihood that a classified object matches the ground situation. The producer’s accuracy is the proportion of object types that were correctly classified. Furthermore, the area proportion was considered to adjust the accuracy assessment results and evaluate the uncertainties of the forest products’ accuracies and area estimates [59], based on a 95% confidence interval. The performance of the accuracy assessment was carried out with ArcGIS 10.0 [51].

### 2.7. Comparison between HJ-1-Based Forest Map and Other Land Cover Products

To assess the forest cover classification of Northeast China, the forest map resulting from this study was compared with two global land cover products: (1) GlobCover 2009 [60], and (2) MCD12Q1 2009 [61]. The detailed information about these two land cover products is presented in Table 2 and Table 3 along with that of the product derived from this study.

The GlobCover 2009 product is available at its official website (http://ionia1.esrin.esa.int/index.asp). The MODIS MCD12Q1 land cover product has several land cover classification schemes, and the primary land cover scheme from the International Geosphere Biosphere Program (IGBP) was used in this study, which is available at the USGS website (https://lpdaac.usgs.gov).

As the MCD12Q1 land cover products use similar forest classification schemes to those for the HJ-1-based forest map (Table 3), not only the total forest area and spatial distribution were compared, but also the extents and quantities of different forest types were assessed in Northeast China. The GlobCover 2009 product has an obviously different forest classification scheme with eight forest-dominated types being combined into one forest layer, and the comparison between this product and the derived HJ-1-based forest map was made only for the total area and the spatial distribution of forest.

In addition, the forest area records for the entirety of Northeast China were available by referencing provincial level agricultural census data. These data were documented in the statistical yearbooks of Jilin Province, Liaoning Province, Heilongjiang Province, and the Inner Mongolia Autonomous Region, and were compared with the forest area estimated from the HJ-1-based forest map.

## 3. Analyses and Results

### 3.1. Forest Distribution of Northeast China Derived from HJ-1 Images

The confusion matrix is presented in Table 4, including user’s accuracy, producer’s accuracy, and overall accuracy of various forest maps in Northeast China. An overall accuracy of 0.91 ± 0.01, with a 95% confidence interval, was obtained from the HJ-1 based forest map. The producer’s accuracy and user’s accuracy of forest were 0.87 ± 0.005 and 0.89 ± 0.02, respectively. The HJ-1 based forest map was in high accuracy. The classification accuracy of forest types was also assessed using the field validation data, indicating that the overall accuracy is approximately 0.75 ± 0.01, with a 95% confidence interval (Table 5). The producer’s accuracy and user’s accuracy of deciduous broadleaf forest were the highest among all types of forest. The accuracies for shrubland and mixed forest were lower than other forest types, primarily due to the complicated mixing of different forest types and limited spatial resolution of remote sensing imagery.

**Figure 5:**
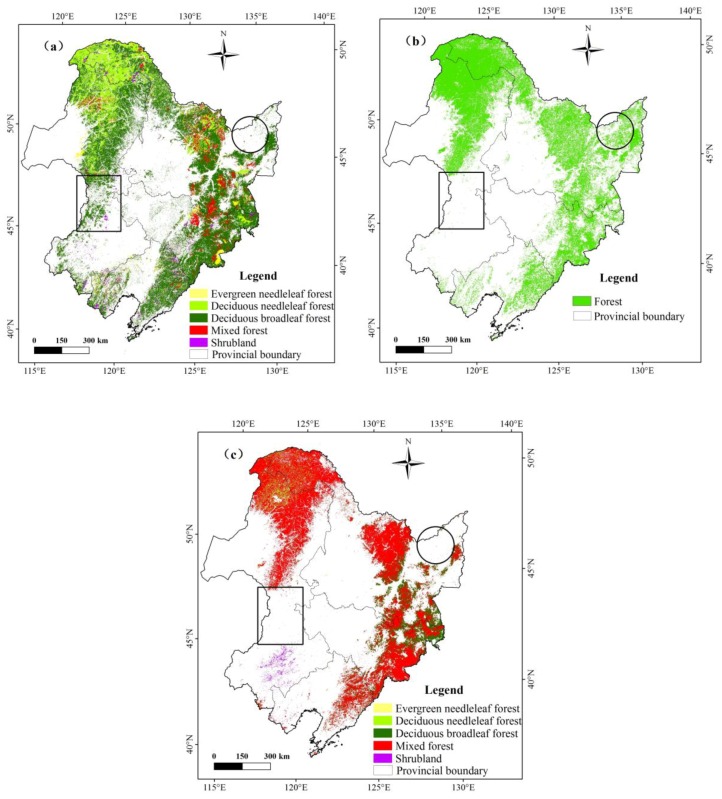
Spatial distribution of forest area from three datasets in Northeast China: (**a**) HJ-1-based forest 2010, (**b**) GlobCover 2009, and (**c**) MCD12Q1 2009.

The forest area was estimated to be 5.0 × 10^5^ km^2^ for Northeast China, accounting for 40.3% of the entire land area in the region (Figure 5a). The forested areas were dominated by deciduous broadleaf forest, which occupied 69% of the forested area, and deciduous needleleaf forest, which is 20% of the forested area. The other forest such as mixed forest, evergreen needleleaf forest, and shrubland is around 7%, 3% and 1% of the forested area, which shows a small proportion of the forest areas. The dominant tree species in Northeast China include Larix gmelinii, Korean pine, spruce, fir, birch, Mongolian oak, lime tree Huang Bo, walnut, Chinese catalpa, elm and maple tree, etc.

**Figure 6:**
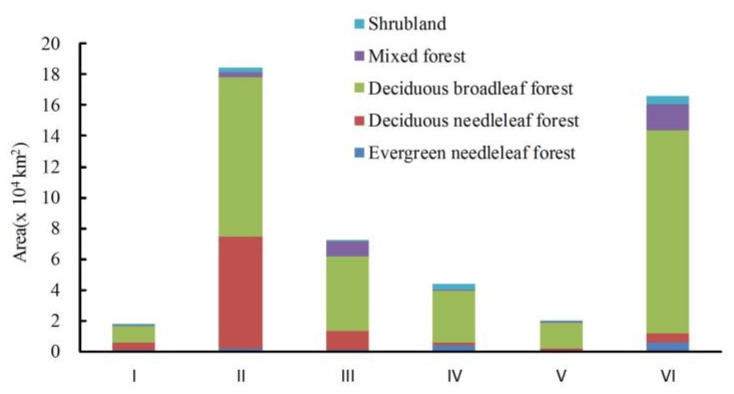
Forest area of ecological functional zones from HJ-1-based imagery in Northeast China. I: Hulun Buir region; II: Daxing’an Mountains; III: Xiaoxing’an Mountains; IV: Songliao Plain; V: Sanjiang Plain; VI: Changbai Mountains.

The areas of all kinds of forest in different ecological functional zones of Northeast China are listed in Figure 6. Forests are largely distributed across mountainous terrain, including the Daxing’an Mountains, Xiaoxing’an Mountains and Changbai Mountains, with limited distribution in the low hills and plains (Figure 5a). The statistical results (Figure 6) also reveal the following order of estimated forest area: Daxing’an Mountains > Changbai Mountains > Xiaoxing’an Mountains > Songliao Plain > Sanjiang Plain > Hulun Buir region. In each sub-region, deciduous broadleaf forest accounted for the largest proportion among all types of forest, and deciduous needleleaf forest comprised the second largest area. Shrubland and evergreen needleleaf forest made up a relatively smaller proportion of all forested areas in each sub-region.

### 3.2. Area Comparison between HJ-1-Based Forest Map and Other Global Products

The estimated forest area from the three forest maps (HJ-1 2010, GlobCover 2009, and MCD12Q1 2009) is listed in Table 6. Among the three maps, the estimated forest area in Northeast China ranged from 4.25 × 10^5^ km^2^ (MCD12Q1 2009) to 5.0 × 10^5^ km^2^ (HJ-1 2010). The estimated forest area from the MCD12Q1 2009 data and the GlobCover 2009 data were both substantially lower than that of the HJ-1 2010 and national statistics data for forested area (NSF 2010, 4.98 × 10^5^ km^2^). The forest proportion (40.3%), classified from the HJ-1-based map is more consistent with the forest coverage (40.22%) disseminated by the Ministry of Environmental Protection of the People’s Republic of China [62].

At the provincial level, Heilongjiang had the largest forest area among the four provinces, whereas Liaoning had the smallest forest area (Table 6). The estimated forest area from the MCD12Q1 2009 data in Heilongjiang was slightly lower than that from the HJ-1 2010, GlobCover 2009, and NSF 2010 data. The difference between HJ-1 2010 and NSF 2010 was smallest in the forest area of Heilongjiang province, compared with other datasets. The forest area in Jilin and Inner Mongolia from the MCD12Q1 2009 data was the smallest compared with other datasets and the differences between MCD12Q1 2009 and GlobCover 2009, HJ-1 2010, and NSF 2010 for the forest area of Jilin province are significant. The forest area of Liaoning from the GlobCover 2009 dataset was the smallest among all the forest maps and the forest area from the MCD12Q1 2009 and HJ-1 2010 datasets was approximately twice that of the GlobCover 2009 dataset. The HJ-1-based forest estimates in the four provinces were much closer to the NSF 2010 results and the difference in the total forest area in Northeast China between these two datasets was only 5038 km^2^, 1% of the total forest area in the NSF 2010 dataset.

### 3.3. Spatial Differences between the HJ-1-Based Forest Map, GlobCover, and MCD12Q1

**Figure 7:**
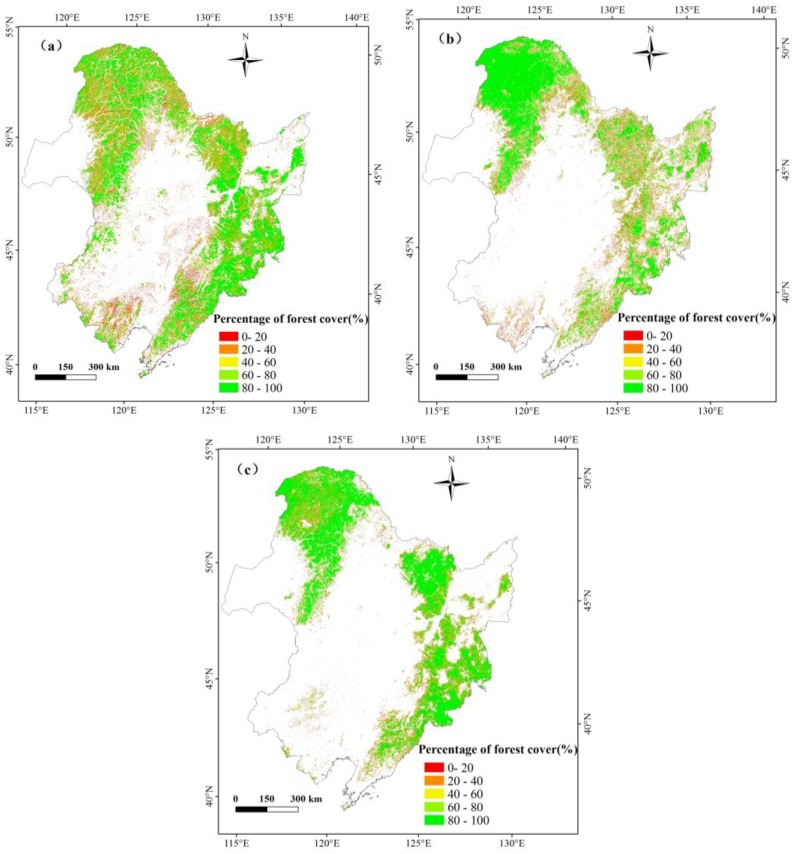
Forest cover percentage maps from HJ-1 2010 (**a**), GlobCover 2009 (**b**) and MCD12Q1 2009 (**c**) at a resolution of 1500 m.

**Figure 8:**
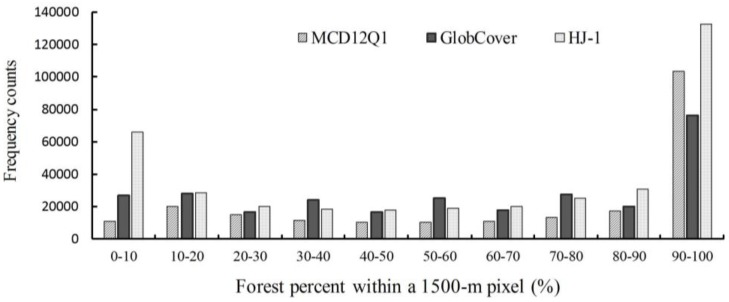
Pixel frequency of different forest percentage from the HJ-1 2010, GlobCover 2009 and MCD12Q1 2009 datasets.

The forest cover percentages from the three datasets (HJ-1 2010, GlobCover 2009, and MCD12Q1 2009) were calculated using a 1500-m resolution aggregated grid (Figure 7); Figure 8 presents a comparison of these datasets represented as pixel frequencies of different forest cover percentages. The histogram reveals that there are approximately three to six times more pixels in the HJ-1 2010 dataset than in the MCD12Q1 2009 and GlobCover 2009 datasets with forest percentages of less than 10%. This is attributed to the fact that HJ-1 imagery at 30-m resolution can be used to identify small patches of forest, but the MCD12Q1 imagery at 500-m resolution and GlobCover imagery at 300-m resolution tend to miss small forest patches. With forest percentages of 90–100%, the pixel counts of the three datasets are all much greater than other forest percentage levels, especially the MCD12Q1 2009 dataset, which accounts for approximately 50% of the total number of pixels contributing to this percentage level. For the percentage ranges of 0–10%, 10–20%, 20–30%, 40–50%, 60–70%, and 80–90%, the order of pixel counts is MCD12Q1 2009 < GlobCover 2009 < HJ-1 2010. The GlobCover 2009 has the largest number of pixel counts within the percentage of 30–40%, 50–60%, 70–80%.

**Figure 9:**
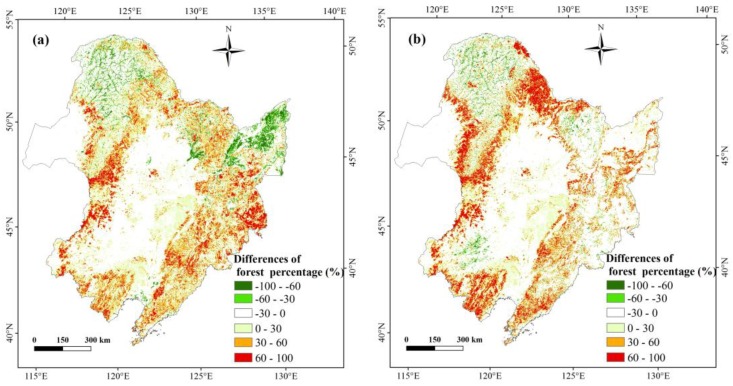
Comparison maps of forest percentage at a spatial resolution of 1500 m between HJ-1 2010 and GlobCover 2009 (**a**) and HJ-1 2010 and MCD12Q1 (**b**); red indicates larger HJ-1 2010 data values, whereas green indicates larger of the other data values.

**Figure 10:**
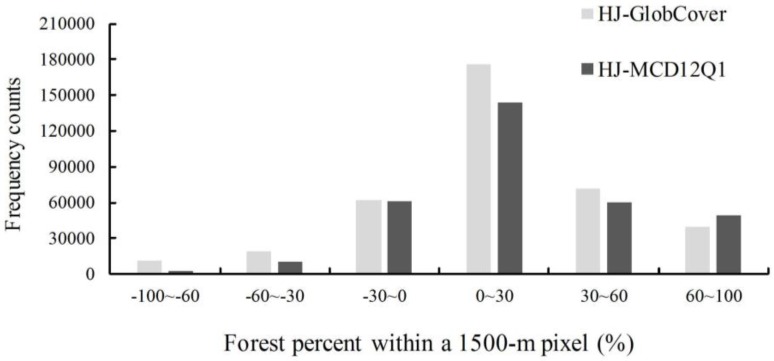
Pixel frequency at various levels of percentage difference between HJ-1 2010 and GlobCover 2009, HJ-1 2010 and MCD12Q1 2009.

To compare the spatial difference at the pixel level, the difference for each of two forest percentage map groupings was calculated (Figure 9 and Figure 10). For the two groupings, more than 75% of the grid cells fall within 0–100% discrepancies across the entire study area except for the northern Daxing’an Mountains, Hulun Buir region, Sanjiang Plain, and Songliao Plain. Approximately 50% of the pixels in the region fall within 0–30% discrepancies, mostly in the Songliao Plain. This indicates that the estimated forest area based on the HJ-1 2010 classification map is higher than that in the MCD12Q1 2009 and GlobCover 2009 datasets, especially for small forest patches. This is because 30-m HJ-1 images can extract small forest patches, whereas MODIS (500-m) and MERIS (300-m) images cannot.

**Figure 11:**
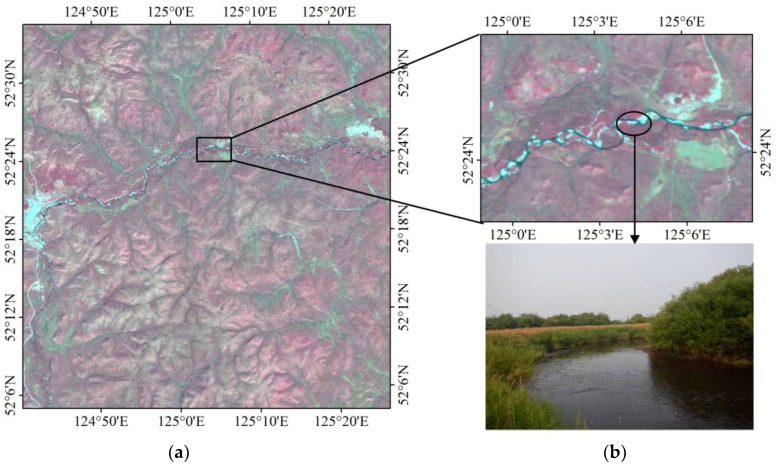
False color image of HJ-1 and a field photo collected from the Daxing’an Mountains. (**a**) Daxing’an Mountains; (**b**) rivers and wetlands.

**Figure 12:**
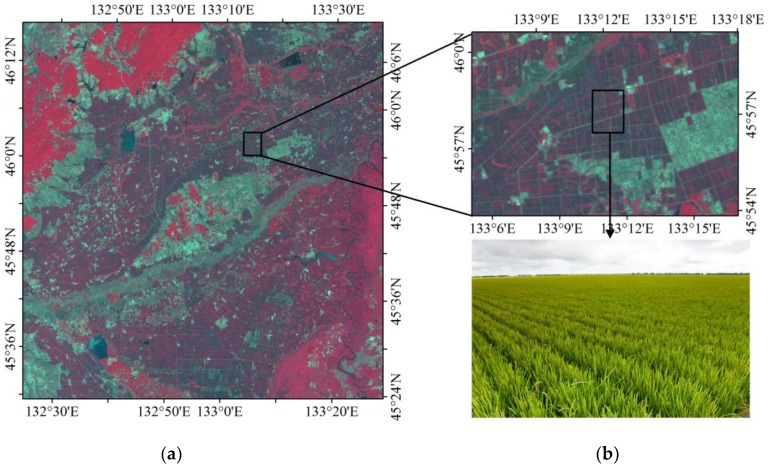
False color image of HJ-1 and a field photo collected from the Sanjiang Plain. (**a**) Sanjiang Plain; (**b**) Cropland.

For the HJ-1 and GlobCover grouping, approximately 8% of the grid cells fall within−30%~−100% of the discrepancies, primarily in the northern Daxing’an Mountain and across the Sanjiang Plain (Figure 9a). In the northern Daxing’an Mountain, the GlobCover map contains larger forest areas than the HJ-1 map, where extensive rivers and wetlands are dispersed across the valleys, as reflected by the shape features of the forest percentage difference between the HJ-1 map and GlobCover map. In the Sanjiang Plain, a large amount of cropland was previously classified as forest. The field samples collected for those intricate regions support the results of the HJ-1 classification (Figure 11 and Figure 12).

For the HJ-1 and MCD12Q1 grouping, approximately 4% of the grid cells fall within −30%~–100% of the discrepancies, primarily in the northern Daxing’an Mountains and across the southwestern Songliao Plain. The MODIS derived map (Figure 5c) shows that there is a relatively large shrub area in the southwestern Songliao Plain, but in the HJ-1-derived map the shrub patches are much smaller and more fragmented.

## 4. Discussion

### 4.1. Forest Classification in Northeast China

The three forest maps demonstrate some degree of spatial consistency for most of Northeast China. However, obvious differences exist among these maps. For example, the HJ-1 2010 and MCD12Q1 maps depict a small forest area for the Sanjiang Plain (the circle in Figure 5), but the GlobCover map shows a larger forest area. The Sanjiang Plain was famous for its large natural wetlands area before the middle of the 20th century, but it is now dominated by cropland [63]. The training samples and validation samples collected from the Sanjiang Plain were used to verify the forest classification, and the results proved that a large area of cropland was present (Figure 12), supporting the results in the HJ-1 2010 and MCD12Q1 maps. In the south of the Inner Mongolia Autonomous Region (the square in Figure 5), the HJ-1-based map contains a larger forest area, but both the GlobCover 2009 and MCD12Q1 2009 maps illustrate a small area of forest. By comparing the results with Google Earth images and the field samples in multiple locations, it is suggested that forest was indeed distributed in the low hilly terrain within the square region. This indicates that HJ-1 imagery has an improved capacity to map forest areas, which can be attributed to its higher spatial resolution than moderate resolution imagery (MERIS and MODIS).

The spatial disagreement of forest from the HJ-1, MCD12Q1, and GlobCover datasets inevitably led to the differences in forest area estimates. The total area of forest from HJ-1 is 18.3% and 11.8% higher than that from MCD12Q1 and GlobCover datasets, respectively. Furthermore, there is large variability in the estimated area across different forest types using HJ-1 imagery (30-m) and MODIS (500-m). MCD12Q1 land cover map indicates that approximately 65% of the forest in the study area is mixed forest (275,720 km^2^), while deciduous broadleaf forest (55,381 km^2^) is only approximately 13% of the total forest area. The HJ-1 results demonstrate that the dominant forest type is deciduous broadleaf forest (344,913 km^2^), comprising approximately 69% of the total forest area. It is worth comparing the results with those by other researchers who have classified the forest types in Northeast China using remote sensing data. Xiao et al. [5] mapped the forest of Northeast China using multi-temporal SPOT-4 VEGETATION sensor data (1-km resolution), determining that the total forest area was 478,287 km^2^, with deciduous broadleaf forest (321,734 km^2^) being the dominant forest type, covering approximately 67% of the total forest area. Whereas this is consistent with the HJ-1 2010 dataset, the mixed forest area (97,071 km^2^) estimated by Xiao et al. [5] is notably larger. Jin [18] used MODIS/NDVI time-series datasets and DEM to classify different forest types and determined that the total forest area of Northeast China was 546,374.53 km^2^, of which approximately 55% is comprised of deciduous broadleaf forest, but mixed forest was not a dominant forest type in this region. However, Yan et al. [19] classified forest subcategories in Northeast China (not including the part of Inner Mongolia) using MODIS-derived NDVI and EVI time series, and the forested regions were dominated by temperate coniferous forest and mixed forest. Furthermore, the land cover data of Northeast China issued from the Data Sharing Infrastructure of Earth System Science (http://www.geodata.cn) were collected, which were derived from Landsat TM/ETM+ and MODIS (250-m) in 2005. The total forest area presented by this dataset is 507,362 km^2^, the dominant forest type is deciduous broadleaf forest, which accounts for 48% of the total forest area, and mixed forest is approximately 18% of the total forest area.

The aforementioned comparison implies that accurately mapping forest is very difficult [52], and there is still great uncertainty in the spatial extent, distribution, and subcategories of forest in Northeast China. Since 2008, the Landsat data freely released by the United States Geological Survey (USGS) has been the major data source for forest mapping with moderate resolution, but HJ-1 images also showed a reliable alternative or supplementary in forest mapping and subcategories identifying in this study. Similar free and open data policies would enable greater use of these data for public good and foster greater transparency of the development, implementation, and reactions to policy initiatives that affect the world’s forests [64]. In the future, how to integrate spectral data from Landsat-like images and structural data from radar observations (e.g., PALSAR and Sentinel-1) to improve the accuracy of forest maps should be considered a priority [65]. On the other hand, to reduce uncertainties in estimating the forest cover in large areas, fine-scale estimates are needed because the rapid land-cover changes related to forest can be observed at a small-scale. UAVs (unmanned aerial vehicles), LiDAR (light detection and ranging), and hyperspectral digital image data sources can provide accurate estimations of many key forest parameters. Results based on the integration of multi-source data have been shown to be superior to results obtained using a single data source [66], and they could provide important validation data for large scale estimation, but the costs of those data sources are higher.

### 4.2. Advantages of Forest Classification Using HJ-1 Images and Object-Based Method

The HJ-1-based forest mapping in this study has obvious advantages, including high spatial resolution and high temporal resolution (2-days). The HJ-1-based forest map in Figure 7 provides detailed fragmentation of the forest in most plains and hilly areas, which is ascribed to the fine resolution of HJ-1 imagery. Many previous studies about forest mapping associated with times series phenology are often supported by high-temporal-resolution sensors images, like AVHRR and MODIS, and moderate-to-high sensors images, like SPOT and Landsat. However, these data have either too low spatial resolution or too low temporal resolution to work at a regional scale. High temporal resolution makes HJ-1 imagery feasible to derive NDVI, NDWI and EVI time series data and to highlight significant seasonal differences among various forest types, which is very useful for separating areas dominated by evergreen tree species from forest dominated by deciduous tree species.

Although forest versus non-forest maps can be extracted automatically or semi-automatically from images over some mountainous terrains, visual interpretation of satellite imagery is still the primary means to do this in the very rugged areas [52]. It is a very tedious and time-consuming process and the accuracy of results largely depends on the experience of professionals. Hence, the visual interpretation is difficult to be repeated over a large area. To map forest cover on a large scale with Landsat-like images, it is necessary to utilize automated classification routines as much as possible to save human resources [67]. In this study, a simple object-based decision tree algorithm was developed to carry out a rapid regional forest mapping. We found that the combination of object-based image analysis and decision tree classification was an effective method of classifying forest across a large area and is accurate based on comparison to ground truth data and global products. Utilizing a decision tree provides a transparent, repeatable, and easily interpreted process to facilitate regular updates and assessments of forest area and their spatial distribution [33]. Forest subcategory classification requires multi-temporal data to incorporate greater spectral variability within each class. Multi-resolution segmentation within the context of object-based classification has been proved to be useful for this more complex discrimination of forest. This method was straightforward and easy to extend to other large-scale regions. The rules created in this study only utilized a few variables, but through further testing and implementation this process may become increasingly automated and accurate.

### 4.3. Uncertainties of Forest Classification in Northeast China

Similarly to other forest classifications, error and uncertainty cannot be avoided in the HJ-1-based forest map produced in this study. One source of uncertainty is how to define individual forest types and describe their characteristics with remote sensing images. Concerning the forest cover in Northeast China, this study sought to determine which forest type is dominant, mixed or deciduous broadleaf forest. This study used the scheme of forest types modified from the IPCC. Deciduous broadleaf forest refers to natural or semi-natural vegetation with a tree canopy cover of more than 15% and tree height between 3 and 30 m. Mixed forest refers to natural or semi-natural vegetation with a tree canopy cover of more than 15% and tree height between 3 and 30 m, in addition to a ratio of needleleaf forest and broadleaf forest that is between 25% and 75%. Notably, MCD12Q1 and GlobCover use different forest definitions [68,69]. MCD12Q1 uses 60% as the low limit of tree canopy cover, whereas GlobCover uses 15%; and the tree height threshold used in MCD12Q1 was 2 m, whereas that in GlobCover was 5 m. For forest cover mapping at large areas, these two factors (tree canopy cover and tree height) are difficult to accurately measure with moderate resolution optical remote sensing data [16]. The important difference between mixed forest and deciduous broadleaf forest is the ratio of needleleaf forest and broadleaf forest. However, the extraction of forest cover from remote sensing is based on objects or pixels, and it is difficult to identify the area ratio of needleleaf to broadleaf forest within a homogeneous area. Therefore, the discrimination of mixed forest and deciduous broadleaf forest primarily depends on the knowledge of experts and the small spectral difference between these forest types in remote sensing images. This naturally leads to another source of uncertainty, i.e., the assessment of accuracy.

A large number of field survey samples were applied to perform the accuracy assessment of the HJ-1-based forest map in this study. However, determining the ratio of needleleaf to broadleaf forest largely relies on the subjective judgments of the field survey participants. This validation dataset collection was performed simultaneously by different field survey groups, implying the same forest type may have been identified as a different forest type, especially, deciduous broadleaf forest and mixed forest. Deciduous broadleaf forest, which is dominated by deciduous broadleaf species, also includes needleleaf species, but the quantity of needleleaf forest species is less than that for mixed forest. When only a small portion of forest is captured and viewed, the forest type can be misclassified. To avoid this problem, it is better to have the same survey group to classify and verify the same region. Furthermore, the training data and validation data were both collected in 2011, while the images used in this study were acquired in 2010. The inconsistency of the data acquisition time may lead to minor uncertainties of estimation. Although in one year the forest land may not have great area changes, the field survey samples used for training and validation should be selected from large, homogeneous forest patches combined with remote sensing images. In addition, the validation data were provided by individual provincial environmental protection departments of Northeast China. The spatial distribution of these field survey samples is even more than the training data which were collected by our research teams. Theoretically, the classification algorithm was influenced by the richness, spatial distribution, and spatial representativeness of the training samples. Because of limits on time, human-resources, and financial support, the training data was only obtained from the positions with convenient transportation. The precision of the estimates in no training data region may be lower, especially relevant to forest types.

The segmentation scale is also a source of uncertainty in this study. Segmentation scales greatly affect the size of objects and the resultant forest cover map. If the segmentation scale is too small, the objects may be highly fragmented. If the segmentation scale is too large, some detailed information may be ignored. In this study, multi-resolution segmentation was applied, with the goal of improving accuracy of forest subcategories classification and reducing the data redundancy. In future, choosing a segmentation scale based on statistics can be tested.

## 5. Conclusions

Existing efforts on large-scale forest mapping generally focus on the use of high temporal and spatial resolution datasets, and our knowledge is still limited in the new frontier of forest shrinking in Northeast China. Based on the object-based decision tree classification approach and all the available HJ-1 images in a single year, this study generated an unprecedented 30-m forest map (including subcategories) in Northeast China in 2010. To our knowledge, this is the first application of HJ-1 imagery on large area (>10,000 km^2^) forest mapping. Its effective implementation in large areas with hundreds of HJ-1 images showcased potentials and prospects of the application of HJ-1 imagery in global and longer temporal scale of land cover and land use changes studies.

In this study, an object-based decision tree classification approach has been proved to be effective in classifying forest across a large area and was accurate based on validation using ground truth data from field surveys. The results also showed better consistency with the statistical data when compared with the two global land cover products (MCD12Q1 and GlobCover). More importantly, HJ-1 imagery provides intensive observations for the phenology attributes of different forest types. This study demonstrates that HJ-1 imagery can reasonably meet the high temporal observation requirement of the forest subcategories mapping in temperate regions. That suggests HJ-1 imagery is promising in the applications of more detailed land use mapping such as different crop planting types and forest species. Furthermore, comparing the results of the HJ-1 forest map to another global land cover product (MCD12Q1) revealed that the forest type information from the global land cover products should be cautiously used in various ecological models. If MCD12Q1 is used in carbon cycle modeling, large uncertainty can be introduced in the carbon evaluation at regional scales. Considering that HJ-1 data only cover China and some surrounding Asian countries, but not the whole globe, and the sensors only have four wave bands; more sources of remote sensing data should be included, such as SAR (Sentinel-1, PALSAR, ENVISAT-ASAR, etc.), LiDAR, UAV, and high temporal resolution optical remote sensing data (Gaofen series satellite, Landsat 8, etc.) for monitoring forest on a global scale. This approach is expected to gain sufficient robustness and reliability on larger scales of forest remote sensing.

## Figures and Tables

**Table 1:** Multi-resolution segmentation parameters used in this study.

Weight	Levels	
	Level 1	Level 2
Scale (Units: pixels/meters)	30/900	10/300
Color	0.7	0.9
Shape	0.3	0.1
Smooth	0.4	0.4
Compact	0.6	0.6

**Table 2:** Characteristics of land cover datasets used in this study.

Product Characteristics	GlobCover (2009)	MODIS Land Cover Type (MCD12Q1)	This Study (2010)
Sensor	MERIS	MODIS	HJ-1 CCD
Time of Data collection	Jan.–Dec. 2009	2010	2010
Classification scheme	UN LCCS (22 classes)	IGBP (17 classes)	IPCC modified (12 classes)
Spatial resolution	300 m	500 m	30 m

**Table 3:** Forest category of GlobCover, MCD12Q1 and this study.

Forest Category from GlobCover (2009)	Forest Category from MCD12Q1 (2009)	Forest Category in This Study (2010)
Closed to open (>15%) broadleaved evergreen or semi-deciduous forest (>5 m)	Evergreen needleleaf forest	Evergreen needleleaf forest
Closed (>40%) broadleave deciduous forest (>5 m)	Evergreen broadleaf forest	Evergreen broadleaf forest
Open (15–40%) broadleaved deciduous forest/woodland (>5 m)	Deciduous needleleaf forest	Deciduous needleleaf forest
Closed (>40%) needleleaved evergreen forest (>5 m)	Deciduous broadleaf forest	Deciduous broadleaf forest
Open (15–40%) needleleaved deciduous or evergreen forest (>5 m)	Mixed forest	Mixed forest
Closed to open (>15%) mixed broadleaved and needleleaved forest (>5 m)	Open shrubland	Shrubland
Mosaic forest or shrubland (50–70%)/grassland (20–50%)	Closed shrubland	
Closed to open shrubland		
Closed to open (>15%) broadleaved forest regularly flooded (semi-permanently or temperately)—Fresh or brackish water		

**Table 4:** Confusion matrix of accuracy assessments of the three forest maps with a 95% confidence interval.

Data		GT Samples		WI	UA	PA	OA
		Forest	Non-Forest				
HJ-1 CCD	forest	1169	142	0.40	0.89 ± 0.02	0.87 ± 0.005	0.91 ± 0.01
	non-forest	359	2303	0.60	0.94 ± 0.01	0.92 ± 0.005	
MCD12Q1	forest	520	127	0.34	0.80 ± 0.03	0.58 ± 0.007	0.71 ± 0.01
	non-forest	1008	2318	0.66	0.70 ± 0.02	0.87 ± 0.007	
GlobCover	forest	555	275	0.36	0.67 ± 0.03	0.56 ± 0.008	0.69 ± 0.02
	non-forest	973	2170	0.64	0.70 ± 0.02	0.79 ± 0.008	

Note: GT: Ground Truth; WI: proportion of area mapped; UA: User’s Accuracy; PA: Producer’s Accuracy; OA: Overall accuracy.

**Table 5:** Confusion matrix of the forest subcategories from HJ-1-based map with a 95% confidence interval.

Class	GT Samples					WI	UA	PA
	ENF	DNF	DBF	MF	SRF			
ENF	84	5	12	3	1	0.03	0.80 ± 0.03	0.76 ± 0.005
DNF	8	100	10	6	4	0.20	0.78 ± 0.04	0.75 ± 0.005
DBF	12	10	213	10	8	0.68	0.84 ± 0.02	0.84 ± 0.004
MF	5	13	16	82	6	0.07	0.70 ± 0.02	0.69 ± 0.008
SRF	1	5	3	17	31	0.02	0.54 ± 0.03	0.62 ± 0.007

Note: GT: Ground Truth; WI: proportion of area mapped; UA: User’s Accuracy; PA: Producer’s Accuracy; ENF: Evergreen needleleaf forest; DNF: Deciduous needleleaf forest; DBF: Deciduous broadleaf forest; MF: Mixed forest; SRF: Shrubland.

**Table 6:** Provincial estimates of forest area (km^2^) from the HJ-1-based map, GlobCover 2009 and MCD12Q1 2009.

	Heilongjiang	Jilin	Liaoning	Inner Mongolia	Total
Evergreen needleleaf forest	4277	4000	3911	2532	14,720
Deciduous needleleaf forest	46,731	2358	1854	47,226	98,169
Deciduous broadleaf forest	126,400	67,198	49,135	102,180	344,913
Mixed forest	20,116	9769	698	2781	33,364
Shrubland	946	1593	5508	3882	11,929
Total HJ-1-based forest area	198,470	84,918	61,106	158,601	503,095
Total MCD12Q1 forest area	187,474	33,258	74,735	129,800	425,267
Total GlobCover forest area	221,999	51,052	32,477	144,328	449,856
National statistics of forest area (NSF)	205,300 ^1^	82,780 ^1^	56,990 ^1^	152,987 [45]	498,057

^1^ from provincial statistics year books (2011).
